# Community sunbed use and photosensitivity reactions: a systematic review

**DOI:** 10.1007/s43630-026-00928-7

**Published:** 2026-06-25

**Authors:** James M. MacLaurin, Kirsty J. Rutter, Lesley E. Rhodes

**Affiliations:** 1 Centre for Dermatology Research, Division of Musculoskeletal and Dermatological Sciences, School of Biological Sciences, Faculty of Biology, Medicine and Health, NIHR Manchester Biomedical Research Centre, The University of Manchester, Manchester, UK; 2 Photobiology Unit, Salford Royal Hospital, NCA NHS Foundation Trust, Manchester Academic Health Science Centre, Salford, Greater Manchester, UK

**Keywords:** Sunbeds, Light-sensitivity, Photosensitivity, Photodermatoses, Drug-induced photosensitivity, Photocontact reactions

## Abstract

Sunbeds emitting ultraviolet radiation (UVR) are an established carcinogen, but little attention has been given to the adverse photosensitivity reactions the UVR exposure can cause. We addressed this information gap through systematic review of the literature on photosensitivity reactions reported following community sunbed use. A systematic search of Medline (via PubMed), CINAHL and Embase identified studies published in English (inception to May 2025). The protocol was registered in Prospero (CRD420251048551). We included all study designs. Quality was assessed using JBI Critical Appraisal Checklists (Case-Reports, Case-Series) and Downes Appraisal (Cross-Sectional Studies), and narrative synthesis performed. Of 413 articles screened, 34 met inclusion criteria (26 case-reports, 6 case-series, 2 cross-sectional studies; 28 high quality for their study design). There were 67 photosensitivity reactions, including 33 cases of photodermatoses (29 pseudoporphyria; 4 solar urticaria, 3 as first presentations), and 3 cases of photoaggravated disorders (one first presentation and two exacerbations of known SLE). There were 31 photosensitivity reactions induced by exogenous substances including 11 to systemic drugs (10 skin, one retinal). Phototoxic reactions occurred to celery, parsnip and lime juice (14 via contact, one ingested), and 5 photocontact reactions to other topical agents including a well-being product. Severe systemic reactions occurred, and 6 cases hospitalised. Findings reveal wide-ranging and severe photosensitivity reactions and indicate attention should be given to providing effective information about the risks and triggers of photosensitivity reactions from sunbeds. This includes to sunbed-outlet staff, sunbed-users, patients with disorders that can be UVR-triggered or aggravated, and people taking potentially photosensitising medication.

**Graphical abstract:**

**Figure:**
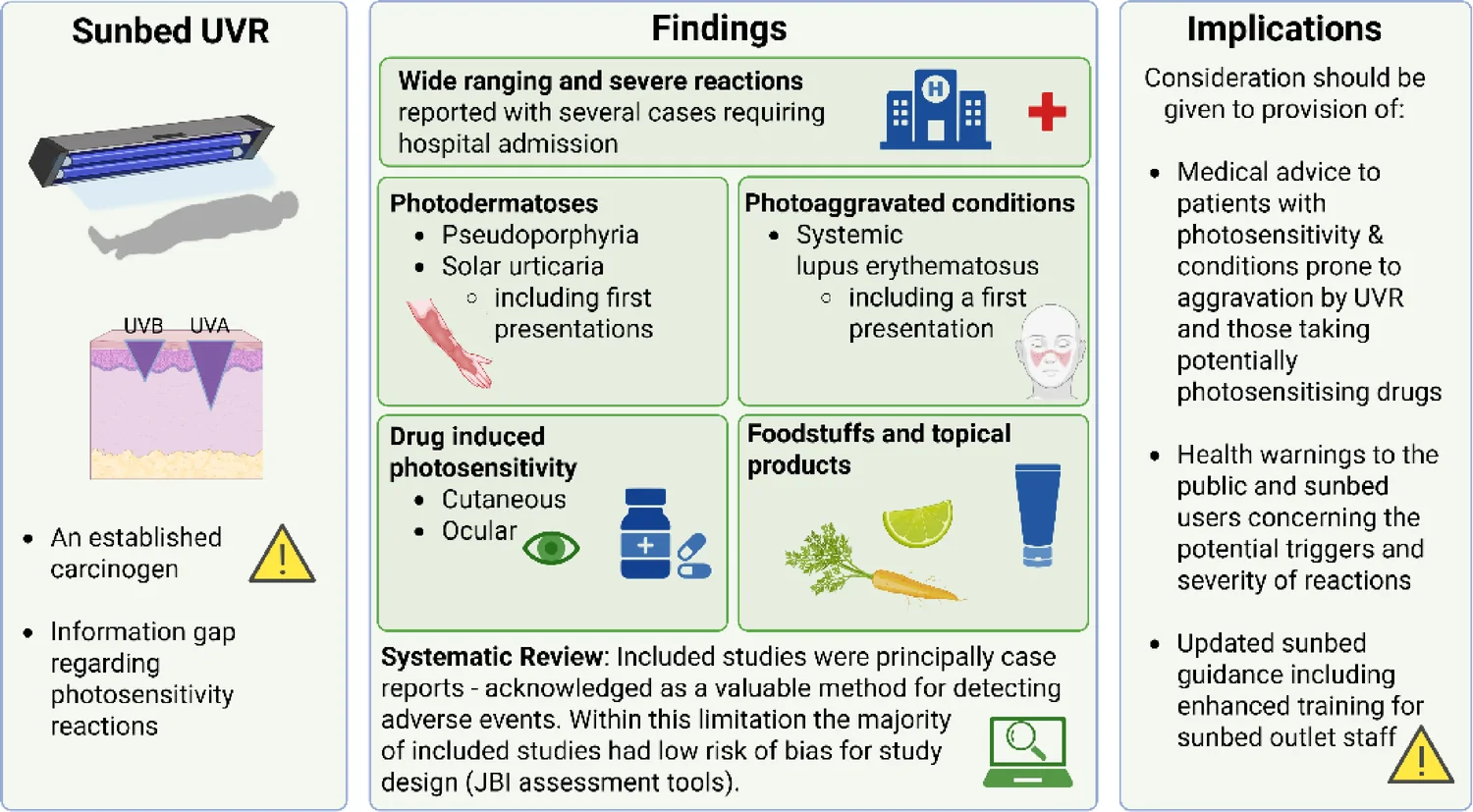
Figure created in BioRender. Rutter, K. (https://BioRender.com/is0qs8d).

## Introduction

Indoor ultraviolet radiation (UVR) tanning devices, commonly known as sunbeds, are in prevalent use despite their classification by the International Agency for Research on Cancer as a first-group carcinogen, through their risks for melanoma and keratinocyte skin cancer [1]. A recent study in 30 European countries found 10.6% population had used a sunbed at least once, with higher prevalence in women [2]. In 2021, there were an estimated ~ 3000 sunbed premises and ~ 18,000 sunbeds in England alone [3]. Despite their established skin cancer risk, little is known regarding adverse photosensitivity reactions occurring following sunbed use.

Sunbeds are found commercially in a range of settings, including hotels, spas, shopping and sports centres [1, 4]. They are categorised in terms of their effective irradiance (W/m^2^) and wavelengths emitted [5]. Type 1 sunbeds do not emit UVB (290–320 nm) and have high UVA irradiance (320–400 nm). Types 2 and 3 emit UVA and UVB, type 2 with relatively high and type 3 relatively low irradiance in the UVA range. Type 4 emit substantially in the UVB range and are limited to medical use [5]. Thus appliances in the community are types 1–3 and primarily emit UVA, although small amounts of UVB are emitted by types 2 and 3. A Swiss survey reported the average UV index (UVI) emitted by sunbeds was 13, i.e. considerably higher than the solar UVI (8.5) at summer midday at intermediate latitudes [6], and a UK survey found 85% sunbeds exceeded the 0.3 W/m^2^ erythemally weighted upper irradiance limit advised for sunbeds in Europe [7, 8]. There are several motivating factors for using sunbeds, including pre-holiday tanning, improving perceived appearance, raising mood, and beliefs such as gaining vitamin D [4], although only UVB-emitting lamps are capable of stimulating vitamin D synthesis [9].

Cutaneous photosensitivity involves abnormal skin reactions to sunlight and artificial sources of UVA, UVB and visible radiation. It can have substantial negative impact, with clinical presentation depending on the specific condition [10, 11]. Photodermatoses include immune-mediated disorders, such as polymorphic light eruption (PLE) and solar urticaria, and biochemical disorders including the cutaneous porphyrias. Pseudoporphyria is similar in appearance to porphyria cutanea tarda (PCT) but with normal porphyrin levels [11–13]. Photoaggravated disorders, i.e. skin conditions exacerbated by UVR, include several connective tissue diseases and inflammatory dermatoses [14]. Photosensitivity also occurs through adverse drug reactions, mostly phototoxic in nature although some through photoallergy [15]. Culprit medication may be prescribed or obtained over-the-counter, and includes many commonly used drugs. Topical agents such as non-steroidal anti-inflammatory drugs (NSAID) and sunscreens can cause photoallergic contact reactions. Photosensitivity reactions may also occur to plant foods/extracts of high psoralen (furocoumarin) content [16], while certain drinks contain quinine, another potent photosensitiser. Eyes are also affected by UVR and while guidance for sunbed-users is to wear protective goggles, surveys repeatedly indicate this may not be followed by sunbed outlets and clients [17]. Adverse ocular effects of UVR include pterygium, cataracts, photokeratitis and conjunctival squamous cell carcinoma [18], while photosensitising drugs have potential to induce ocular phototoxic reactions [19].

Despite these potential complications, there are currently no reviews of photosensitivity reactions occurring with sunbed use. The objective of this work was to address this information gap by performing a comprehensive and systematic review examining the published literature for reports on photosensitivity reactions following sunbed use in the community. This encompasses photosensitivity reactions of skin and eye, and includes photodermatoses, photoaggravated skin disorders and photosensitivity induced by medications, foods and topical agents.

## Methods

### Search strategy and study selection

A systematic search of EMBASE, Medline (via PubMed) and CINAHL was performed for full-text articles published since the inception of each database (~ 1947, 1946, 1937, respectively) until May 8th 2025. Additional studies were identified via reference lists. Searches were conducted according to Preferred Reporting Items for Systematic reviews and Meta-Analysis (PRISMA) guidelines. The review was registered with PROSPERO (CRD420251048551) where the protocol is available.

The following search terms were applied to the databases: (photoderm* OR photosens* OR phototox* OR photoaggrav* OR photoallerg* OR photoreact* OR photoexacerb* OR “light-exacerb*” OR photodermatitis OR “polymorph* light eruption” OR “chronic actinic dermatitis” OR “xeroderma pigmentosum” OR “hydroa vacciniforme” OR porphyria OR pseudoporphyria OR “solar urticaria” OR lupus OR “actinic prurigo” OR “drug-induced” OR “juvenile spring eruption” OR phytophotodermatitis OR “herpes simplex” OR “cold sore” OR photocontact OR genophotodermatos*) AND (“indoor tanning” OR sunbed OR “tanning bed” OR “tanning booth” OR “tanning salon” OR solarium OR solaria OR sunlamp OR “artificial tanning” OR “UV tanning” OR “non-solar ultraviolet radiation” OR “non-solar UV radiation”).

We predefined inclusion criteria as all study types published in the English language which reported photosensitivity reactions including skin and/or eyes following use of community-based sunbeds. Studies focusing on clinic/hospital-based phototherapy reactions were excluded, along with reports of non-photosensitive effects such as skin cancer.

### Data extraction and quality assessment

Titles and abstracts were screened independently by two authors (JMM, KJR); consensus was reached by discussion. Those selected based on the abstract, and articles without abstracts available, underwent full-text review, including review of the references. Studies were selected for inclusion by two authors and confirmed with a third author. Two clinical expert authors (KJR, LER) appraised the photosensitivity diagnoses. Data extracted included study type, presenting clinical features, demographics (age, gender, skin type if available), geographical location, sunbed usage and investigations conducted for diagnostic purposes. Since most included studies were case reports, analysis was by narrative synthesis.

Quality assessment of included studies was performed independently by two authors (KJR, JMM). The methodological quality of case reports and case series was evaluated using the Joanna Briggs Institute (JBI) Critical Appraisal Checklists for Case Reports and Case Series, respectively [20]. These tools evaluate eight and ten aspects of each manuscript respectively, including clarity of reporting on patient characteristics, case description and diagnostic tests. Quality assessment of cross-sectional studies utilised the Downes Appraisal tool for Cross-Sectional Studies (AXIS) [21]. Studies were assessed as high, medium, or low quality based on their quality assessment score; definitions for quality level are shown in each quality assessment table.

## Results

**Fig. 1:**
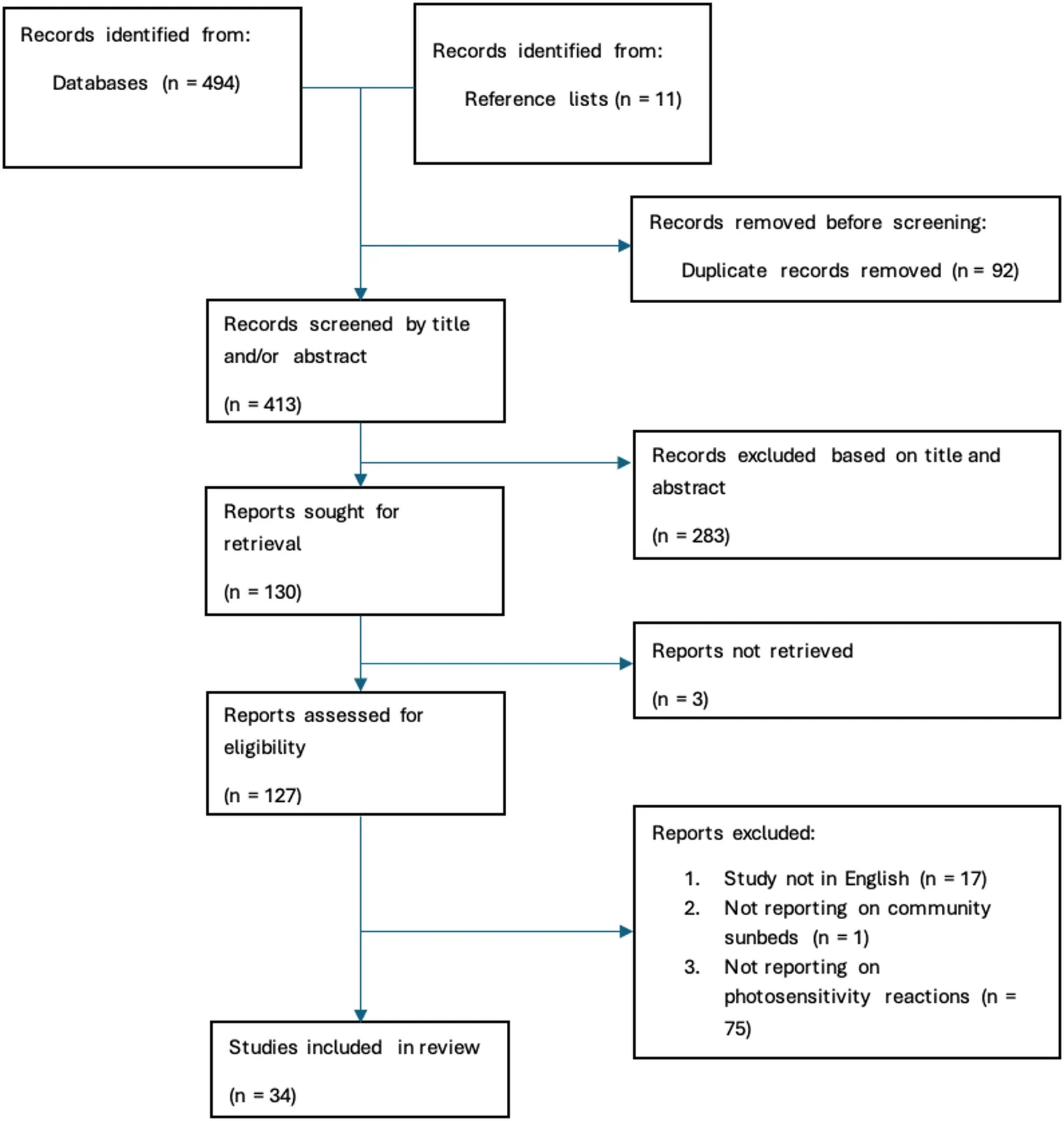
PRISMA flow diagram. The search terms identified 505 records from the three databases including 11 records from their reference lists. The search resulted in the inclusion of 34 studies

The PRISMA flow diagram shows the search results (Fig. 1) [22–55]. Our search identified 505 potentially eligible records. After removal of duplicates, 413 records were screened by title and/or abstract, leading to full review of 127 papers, and resulting in inclusion of 34 records.

### Study characteristics

These were 26 case reports, 6 case series and two cross-sectional studies, published between 1985 and 2024. A total of 67 cases of photosensitivity reactions were reported, comprising 33 cases of primary photodermatoses (Table 1), 3 of photoaggravated disorders (Table 2), 11 of systemic medication induced photosensitivity (Table 3) and 20 cases of cutaneous reactions following contact with or ingestion of other exogenous substances inducing photosensitivity, including foodstuffs (Table 4). The majority of reports provided no information on the specific type of sunbed used. Twelve articles were from the USA, 10 from the UK and 6 from Sweden, with 6 from other countries, i.e. Austria, Colombia, Ireland, Israel, Italy and Turkey. In the case reports/series (*n* = 56 cases), 51 cases were female (91%) and 5 were male (9%). The cross-sectional studies (*n* = 11 cases) did not report sex distribution. Skin phototype was reported in 8 cases overall; all were skin phototype II-III. Twenty-eight studies were assessed as high quality, 3 as medium and 3 as low quality (Tables 5, 6 and 7).

**Table 1:** Summary of the included studies reporting on photodermatoses occurring with community sunbed use

Source	Location	Study design	Age/sex	Type of sunbed	Sunbed usage	Clinical features	Relevant investigations*	Photosensitivity reaction type
Bendsöe et al. [22]	Sweden	Case reports (*n* = 2)	2 F aged 38	UVA	3 visits (case 1); 5 visits (case 2)	Widespread erythema ± wheals, dizzy/faint, syncope in 1 case. Both had subsequent similar symptoms on sunlight exposure.	Phototesting – both had whealing with broadband UVA and UVB.	Solar urticaria
Epstein et al. [23] (Abs)	USA	Case series (*n* = 5)	5 F aged 19–48	UVA	4–9 months of sunbed use for 2–7 days a week	Blistering, fragility and scar formation on dorsal hands (*n* = 5), feet (*n* = 1) and knees (*n* = 1). Medication included thiazide (1), oestrogen replacement (1).	Skin biopsy “compatible with cutaneous porphyria”.	Pseudoporphyria
Esdaile et al. [24]	UK	Case report	M aged 26	N/K	10 year history of sunbed use	Recurrent itchy, red rash only on pre-existing bruises on body, triggered by exposure to sunlight or sunbeds.	Monochromator phototesting on bruises – flare with UVB (310 nm) and with UVA (360 nm). Normal tests on unbruised skin.	Solar urticaria confined to areas of bruising
Farr et al. [25]	UK	Case series (*n* = 6)	5 F aged 17–37, 1 M aged 37	UVA	Regular use ranging from 2–7 sessions per week for 1–6 years; estimated doses 9–77 KJ/cm^2^	Skin fragility; thickened and inelastic skin on dorsal hands; blisters on dorsal hands, feet and shins; hyperpigmented scars and milia. 3 patients were taking oral contraceptive; 1 occasional furosemide.	Skin biopsies (*n* = 4): subepidermal blisters with minimal inflammatory infiltrate Normal erythemal responses to UVB (300 nm) and UVA (350 nm) several months post-sunbed use (*n* = 4).	Pseudoporphyria
Khan et al. [26] (Abs)	UK	Case report	‘Young’ F	N/K	N/K	Tense blisters across arms and legs		Pseudoporphyria
Murphy et al. [27]	UK	Case series (*n* = 4)	4 F aged 28–47	Predominantly UVA	Regular use for 3–15 years	Fragility and blistering of hands, face, or limbs; with scarring in 2 cases; purpuric streaks and excoriations in 1 case. Medication included ibuprofen and coproxamol in 1 case.	Biopsy in 2 cases showed subepidermal blister without inflammation; Monochromator testing normal in 2 cases tested.	Pseudoporphyria
Poh-Fitzpatrick et al. [28]	USA	Case series (*n* = 3)	3 F aged 21–43	UVA (1 case) or UVA and UVB (2 cases)	Regular use ranging from 3–5 times per week, for 8 months – 4 years	All had skin fragility and blisters on dorsal hands, 1 also on feet with scarring; 1 had milia and mild hypertrichosis Medications included analgesics (2), oral contraceptive (1), furosemide (1), antihistamine (1), occasional tetracycline intended to boost tan (1). Use of topical “tanning accelerator” in 2 cases.	Biopsies done in 2 cases: subepidermal bullae consistent with porphyria	Pseudoporphyria
Rademaker et al. [29]	UK	Case series (*n* = 3)	3 F aged 21–27	UVA	Regular use 3–4 times weekly for 12–24 months	Recurrent blistering and scarring of the dorsal hands, lower arms and shins, with fragility, milia and pigmentation. 2 were taking oral contraceptives.	Skin biopsies: subepidermal bullae in all 3 cases	Pseudoporphyria
Ross et al. [30]	UK	Case report	M aged 50s	N/K	2 min on 2 occasions	Widespread erythematous rash, dizziness on both occasions. Loss of consciousness, presumed anoxic seizure from reduced cerebral perfusion.	Normal ECG, EEG, Brain MRI. Prior phototests had confirmed solar urticaria with broad spectrum exquisite photosensitivity (UVB, UVA, visible light)	Exacerbation of solar urticaria
Stenberg et al. [31]	Sweden	Case series (*n* = 5)	5 cases 4 F aged 21–44, 1 M aged 24	N/K	Regular use ranging from 2–5 times weekly for 2-“many” years	Recurrent blisters ± erosions, scarring, and fragility, on dorsal hands and fingers ± lower limbs ± mild hypertrichosis. Medications included oral contraceptive (1), naproxen (2), spironolactone (1), codeine (1)	Biopsy in 3 cases showed subepidermal bullae.	Pseudoporphyria
Wilson et al. [32]	UK	Case reports (*n* = 2)	2 F aged 22 (MZ twins)	UVA	Regular use 3–5 times weekly for 14–24 months	Blistering and scarring on dorsal hands.	Biopsies: subepidermal blisters	Pseudoporphyria

* All cases had investigations in dermatology departments including negative porphyrin screens

*N/K* not known, *Abs* abstract, *M* male, *F* female, *UV* ultraviolet, *MRI* magnetic resonance imaging, *EEG* electroencephalogram, *ECG* electrocardiogram, *MZ* monozygotic

**Table 2:** Summary of the included studies reporting on photoaggravated conditions occurring after community sunbed use

Source	Location	Study design	Age/sex	Type of sunbed	Sunbed usage	Clinical features	Relevant investigations	Photosensitivity reaction type
Fruchter et al. [33]	Israel	Case report	22y F	N/K	3 sessions	Widespread burning, erythematous rash; fever, arthralgia with swelling of ankles, knees and hands, malaise and weight loss.	Raised inflammatory markers. High titre positive ANA; positive anti dsDNA & anticardiolipin antibodies Proteinuria Skin biopsy: urticarial vasculitis	SLE – new diagnosis
Geara et al. [34]	USA	Case report	53y F	N/K	1–2 times weekly for 2 months	Widespread erythematous rash, swollen knees and ankles, fever, possible seizure, incontinence, reduced consciousness. Previously diagnosed stable SLE with skin and joint involvement.	ANA positive. CSF: high protein levels, no infection. EEG: diffuse slowing. CT head normal	Lupus cerebritis; exacerbation of pre-existing SLE
Stern et al. [35]	USA	Case report	30y F	N/K	15 sessions	Extensive erythematous rash on arms, chest and face. Polyarthralgia and morning stiffness. Previously diagnosed SLE	Positive ANA, ENA, RNP. Proteinuria. Elevated white cell count.	SLE exacerbation

*SLE* systemic lupus erythematosus,* N/K* not known,* F* female, *M* male, *y* years of age, *ANA* antinuclear antibodies, *dsDNA* double stranded deoxyribonucleic acid, *CSF* cerebrospinal fluid, *EEG* electroencephalogram, *CT* computed Tomography, *ENA* extractable nuclear antigen, *RNP* ribonucleic protein

**Table 3:** Summary of the included studies reporting on photosensitivity reactions attributed to systemic medication after community sunbed use

Source	Location	Study design	Age/sex	Type of sunbed	Sunbed usage	Clinical features	Drug history/timing	Relevant investigations	Photosensitivity reaction type
Barbosa et al. [36]	USA	Case report	37y F	N/K	Frequent use, including after initiation of voricon-azole	Painful blisters on back, feet and hands; large tense bullae.	Onset one week after commencing oral voriconazole.	Skin biopsy: intraepidermal bullae, spongiosis, oedema and vacuolar interface dermatitis. Negative porphyrin screen, ANA, bullous pemphigoid antibodies.	Bullous phototoxicity from voriconazole
Bilsland et al. [37]	UK	Case report	43y F	UVA	Weekly for 4 months.	Painful blistering and erosions on lower legs, erythema on legs and trunk. Subsequent scarring and milia.	Onset 3 weeks after a 6-day course of oral nalidixic acid	Skin biopsies: subepidermal bulla with minimal cellular infiltrate. Porphyrin screen normal	Pseudoporphyria induced by nalidixic acid
Cohen et al. [38]	USA	Case report	48y F	N/K	Regular use for 5 weeks.	Burning sensation and redness on exposed skin, then oedema and large, painful bullae on hands.	Sunbed use 4 h after taking lomefloxacin; symptom onset soon after use	N/K	Pseudoporphyria induced by lomefloxacin
Costagliola et al. [39]	Italy	Case report	40y F	N/K	N/K, but removed UV-protective eyewear for a few minutes while using.	Blurred vision, reduced acuity with superior central scotoma in left eye. Retinal lesions in both eyes.	Long term hydro-chlorothiazide and lisinopril; symptom onset immediately after sunbed use	Retinal photography: perifoveal autofluorescence and juxtafoveal hypofluorescent zone in right eye. Optical coherence tomography: hyperreflecting thickening in left eye. Suggestive of phototoxic macular damage.	Hydrochlorothiazide induced retinal phototoxicity
Gatson et al. [40]	USA	Case report	22y F	N/K	Single use	Initial mild cutaneous and mucosal reaction to oral ibuprofen; severe exacerbation following sunbed use. Severe erythema and blisters on sunbed exposed sites.	Ibuprofen ingestion; severe exacerbation of symptoms within 24 h of sunbed use	Skin biopsy consistent with TEN	TEN, exacerbated by sunbed exposure.
Nettelblad et al. [41]	Sweden	Case reports (*n* = 2)	22y F	N/K	Single session after ingesting psoralen tablets.	Burning sensation of the skin, progressive blistering ~ 50% BSA, and extensive skin loss requiring skin grafts to 10% BSA.	Oral psoralen tablets x3 (not prescribed) taken prior to sunbed use; symptom onset the following day.	N/K	Psoralen induced burn
			23y F	N/K	Single session after ingesting psoralen tablets	Rapid onset skin irritation, swelling of the skin, blistering of face, arms and legs.	Oral psoralen tablets x6 (not prescribed) taken prior to sunbed use; symptom onset during use.		
Russomanno et al. 2021 [42]	USA	Case report	22y F	N/K	Regular (daily) use	Photodistributed macules and papules with flaccid bullae, plus mucosal involvement.	Onset 10 days after commencing oral lamotrigine	Biopsy: interface dermatitis with scattered dyskeratosis and necrotic keratinocytes, consistent with SJS/TEN	Photodistributed TEN in association with lamotrigine
Sever et al. [43]	Turkey	Case report	17y F	UVA	Unknown	Severe burns, affecting ~ 73% BSA.	Chlorpropamide and oral tetracycline - duration not recorded	Clinical diagnosis	Chlorpropamide and tetracycline induced phototoxic burn
Suarez et al. [44]	USA	Case report	37y F	N/K	1 year	Blisters and fragility on hands and feet, redness, scarring and milia on hands and feet. Resolved on discontinuing naproxen.	Naproxen, danazol, hydrochlorothiazide, hydroxyzine - duration not recorded. Symptom onset around time commenced sunbed use.	Porphyrin screen normal, positive ANA. Skin Biopsy: subepidermal bulla with festooning of dermal papillae and no inflammation.	Pseudoporphyria induced by naproxen
Ting et al. [45]	USA	Case report	53y F	N/K	Weekly use for past 6 months	Widespread red, blistering eruption on exposed sites (sparing sites protected during sunbed use), positive Nikolsky sign.	Daily naproxen; intermittent rabeprazole. Onset 2 weeks after last sunbed use.	Serology– ANA+; Ro+, dsDNA+ Biopsies – subepidermal blistering with full thickness epidermal necrosis and positive immunofluorescence	TEN-like acute cutaneous lupus erythematosus, presumed triggered by oral naproxen and UVR exposure.

*N/K* not known,* M* male,* F* female,* y* years of age,* BSA* body surface area,* ANA* antinuclear antibodies,* UV* ultraviolet,* TEN* toxic epidermal necrolysis,* SJS* Stevens-Johnson syndrome,* dsDNA* double stranded deoxyribonucleic acid

**Table 4:** Summary of the included studies reporting on photosensitivity reactions induced by exogenous foods and topical agents after community sunbed use

Source	Location	Study design	Age/sex	Type of sunbed	Sunbed usage	Clinical features	Exogenous agent history/timing	Relevant workup/investigations	Photosensitivity reaction type
Bang Pedersen et al. [46]	Sweden	Case report (*n* = 2)	2 F aged 61 and 46	UVA	Used sunbeds 3 h after handling parsnips	Painful erythema and blisters on palms and wrists	Peeling or handling parsnips 3 h prior to sunbed use; symptom onset the next day	Clinical diagnoses	Phototoxic reactions to parsnips
Centres for Disease Control [47]	USA	Cross-sectional study (5 sunbed users)	NR	N/K	N/K but noted 5 sunbed users out of 14 affected grocery store employees	Papular, well-circumscribed rash on upper limbs, with residual blistering or hyperpigmentation.	All cases had regular contact with fresh plants at work.	Clinical diagnosis 5/14 (36%) cases were sunbed-users vs. 2/38 (5%) non-cases.	Phytophoto-dermatitis
Eustace et al. [48]	UK	Case report	22y F	N/K	Single session	Streaky erythema, coalescing vesicles and bullae on chest, hands, forearms and thighs.	Spilt large quantities of lime juice on skin at work; sunbed use after work. Symptom onset the next day.	Porphyrin screen normal	Phytophoto-dermatitis induced by spilt lime juice
Geary et al. [49]	UK	Case report	26y F	N/K	Single use after a psoralen bath (non-prescribed)	Burning pain, erythema and blisters on trunk and limbs	8-methoxsalen (psoralen) added to bath water. Symptom onset 19 h after sunbed use.	Clinical diagnosis	Psoralen induced burn
Gilhooley et al. [50]	Ireland	Case report	33y F	N/K	N/K	Cobblestone erythema of lips with crusting.	Cosmetic lip tattoos 18 months previously. Symptom onset followed sunbed use.	Lip biopsy – epithelioid histocytes forming discrete noncaseating granulomas.	Photoinduced granulomatous reaction to tattoo pigment
Hindsen et al. [51]	Sweden	Case report	50y M	N/K	N/K: ‘regular’ use	Eczematous lesions developed on both knees, spreading to his legs and trunk a week later.	Topical ketoprofen gel, and ketoprofen-contaminated bandage. Symptom onset 1 week after sunbed use, on two occasions.	Patch and photo-patch testing – photoallergy to ketoprofen	Photoallergic contact dermatitis induced by ketoprofen
Kaddu et al. [52]	Austria	Case report	41y F	UVA	Single session	Widespread painful, red, oedematous eruption with bullae on the face, neck and limbs.	Exposure to bergamot oil vaporised through heating, just prior to sunbed use. Symptom onset 48–72 h later.	Clinical diagnosis	Phototoxic reaction to bergamot oil
Ljunggren et al. [53]	Sweden	Case report	65y F	UVA	Single session	Generalised burning erythema, large blisters and oedema, and fever. Unexposed sites spared.	Consumed ~ 450 g celery 1 h prior to sunbed use. Symptom onset the following day.	Clinical diagnosis	Phototoxic burn following celery ingestion
Pérez-Camelo et al. [54]	Colombia	Case report	38y F	N/K	Single session	Itching and erythema, progressing to severe burns affecting ~ 85% BSA.	Covered skin with rue, then showered, before using sunbed. Symptom onset the next day.	Clinical diagnosis.	Phytophototoxic reaction to rue extract
Seligman et al. [55]	USA	Cross sectional study (6 sunbed users)	NR	N/K	N/K but 6 sunbed users out of 19 affected grocery store employees	Severe blistering reactions occurred in the sunbed users, erythema and hyperpigmentation	All cases were celery produce handlers	Clinical diagnosis. Reactions in 6/8 (75%) sunbed users vs. 13/39 (33%) non-sunbed-users	Phytophoto-dermatitis

*N/K* not known,* F* female,* M* male,* y* years of age,* NR* not reported

**Table 5:** Quality assessment table using the JBI tool for case reports [20]

Source	1. Were patient’s demographic characteristics clearly described?	2. Was the patient’s history clearly described and presented as a timeline?	3. Was the current clinical condition of the patient on presentation clearly described?	4. Were diagnostic tests or assessment methods and the results clearly described?	5. Was the intervention(s) or treatment procedure(s) clearly described?	6. Was the post-intervention clinical condition clearly described? ^a^	7. Were adverse effects (harms) or unanticipated events identified and described?	8. Does the case report provide takeaway lessons?	Quality Assessment
Bang Pedersen et al. [46]	Yes	Yes	Yes	No	Yes	N/A	Yes	Yes	High
Barbosa et al. [36]	Yes	Yes	Yes	Yes	Yes	N/A	Yes	Yes	High
Bendsöe et al. [22]	Yes	Yes	Yes	Yes	Yes	N/A	Yes	Yes	High
Bilsland et al. [37]	Yes	Yes	Yes	Yes	Yes	N/A	Yes	Yes	High
Cohen et al. [38]	Yes	Yes	Yes	No	Yes	N/A	Yes	Yes	High
Costagliola et al. [39]	Yes	Yes	Yes	Yes	Yes	N/A	Yes	Yes	High
Esdaile et al. [24]	Yes	Yes	Yes	Yes	Yes	N/A	Yes	Yes	High
Eustace et al. [48]	Yes	Yes	Yes	Yes	Yes	N/A	Yes	Yes	High
Fruchter et al. [33]	Yes	Yes	Yes	Yes	Yes	N/A	Yes	Yes	High
Gatson et al. [40]	Yes	Yes	Yes	Yes	Yes	N/A	Yes	Yes	High
Geara et al. [34]	Yes	Yes	Yes	Yes	Yes	N/A	Yes	Yes	High
Geary et al. [49]	Yes	Yes	Yes	Yes	Yes	N/A	Yes	Yes	High
Gilhooley et al. [50]	Yes	Yes	Yes	Yes	No	N/A	Yes	Yes	High
Hindsen et al. 2004 [51]	Yes	No	Yes	Yes	No	N/A	Yes	Yes	Medium
Kaddu et al. [52]	Yes	Yes	Yes	No	Yes	N/A	Yes	Yes	High
Khan et al. (Abs) [26]	No	No	Yes	No	Yes	N/A	Yes	Yes	Low
Ljunggren et al. [53]	Yes	Yes	Yes	Yes	Yes	N/A	Yes	Yes	High
Nettelblad et al. [41]	Yes	Yes	Yes	No	Yes	N/A	Yes	Yes	High
Pèrez-Camelo et al. [54]	Yes	No	Yes	No	Yes	N/A	Yes	Yes	Medium
Ross et al. [30]	Yes	Yes	Yes	Yes	Yes	N/A	Yes	Yes	High
Russomanno et al. [42]	Yes	Yes	Yes	Yes	Yes	N/A	Yes	Yes	High
Sever et al. [43]	Yes	No	Yes	No	Yes	N/A	Yes	Yes	Medium
Stern et al. [35]	Yes	Yes	Yes	Yes	Yes	N/A	Yes	Yes	High
Suarez et al. [44]	Yes	Yes	Yes	Yes	No	N/A	Yes	Yes	High
Ting et al. [45]	Yes	Yes	Yes	Yes	Yes	N/A	Yes	Yes	High
Wilson et al. [32]	Yes	Yes	Yes	Yes	Yes	N/A	Yes	Yes	High

^a^ Question 6 was not scored in this context as it replicated question 3, making it redundant

(Abs) - an abstract only, without full publication. High quality: ≥6 “Yes” answers, Medium quality: 5 “Yes” answers, Low quality: ≤ 4 “Yes” answers

N/A not applicable

**Table 6:** Quality assessment table using the JBI tool for case series [20]

Source	1. Were there clear criteria for inclusion in the case series?	2. Was the condition measured in a standard, reliable way for all participants included in the case series?	3. Were valid methods used for identification of the condition for all participants included in the case series?	4. Did the case series have consecutive inclusion of participants?	5. Did the case series have complete inclusion of participants?	6. Was there clear reporting of the demo-graphics of the participants in the study?	7. Was there clear reporting of clinical information of the participants?	8. Were the outcomes or follow up results of cases clearly reported?	9. Was there clear reporting of the presenting site(s)/clinic(s) demographic information?	10. Was statistical analysis appropriate?	Quality Assessment
Epstein et al. (Abs) [23]	Yes	Unclear	Unclear	Unclear	Unclear	Yes	No	No	Yes	N/A	Low
Farr et al. [25]	Yes	Yes	Yes	Unclear	Unclear	Yes	Yes	Yes	Yes	N/A	High
Murphy et al. [71]	Yes	Yes	Yes	Unclear	Unclear	Yes	Yes	Yes	Yes	N/A	High
Poh-Fitzpatrick et al. [28]	Yes	Yes	Yes	Unclear	Unclear	Yes	Yes	Yes	Yes	N/A	High
Rademaker et al. [29]	Yes	Unclear	Yes	Unclear	Unclear	Yes	Yes	Yes	Yes	N/A	High
Stenberg et al. [31]	Yes	Yes	Yes	Unclear	Unclear	Yes	Yes	Yes	Yes	N/A	High

(Abs) - an abstract, without full publication.

High quality: ≥6 “Yes” answers, Medium quality: 5 “Yes” answers, Low quality: ≤ 4 “Yes” answers

**Table 7:** Quality assessment for the Centres for Disease Control 1985 [47] and Seligman et al. 1987 [55] reports using the AXIS tool for cross-sectional studies [21]

1	Question	Centre for Disease Control 1985 [47]	Seligman et al. 1987 [55]
	Were the aims/objectives of the study clear?	No	Yes
2	Was the study design appropriate for the stated aims?	N/A - investigation post outbreak	Yes
3	Was the sample size justified?	N/A	Yes
4	Was the target/reference population clearly identified?	Yes	Yes
5	Was the sample frame taken from an appropriate population base so that it closely represented the target/reference population under investigation?	Yes	Yes
6	Was the selection process likely to select subjects/participants that were representative of the target/reference population under investigation?	Yes	Yes
7	Were measures undertaken to address and categories non-responders?	NK	Yes
8	Were the risk factor and outcome variables measured appropriate to the aims of the study?	NK	Yes
9	Were the risk factor and outcome variables measured correctly using instruments/measurements that had been trialled, piloted or published previously?	NK	Yes
10	Is it clear what was used to determine statistical significance and/or precision estimates? (e.g. p-values, confidence intervals)	Yes	Yes
11	Were the methods (including statistical methods) sufficiently described to enable them to be repeated?	No	Yes
12	Were the basic data adequately described?	Yes	Yes
13	Does the response rate raise concerns about non-response bias?	No	No
14	If appropriate, was information about non-responders described?	No	Yes
15	Were the results internally consistent?	NK	NK
16	Were the results presented for all the analyses described in the methods?	NK	Yes
	Discussion		
17	Were the authors’ discussions and conclusions justified by the results?	Yes	Yes
18	Were the limitations of the study discussed?	No	Yes
19	Were there any funding sources or conflicts of interest that may affect the authors’ interpretation of the results?	NK	NK
20	Was ethical approval or consent of participations attained?	NK	NK
	Overall quality rating	Low	High

Centres for Disease Control 1985 [47] quality assessment – Low due to 5 “No” answers.

Seligman et al. 1987 [55] quality assessment – High due to 1 “No” answer.

### Photodermatoses

Pseudoporphyria was the most frequent primary photodermatosis (*n* = 29 cases), with the remainder being solar urticaria (*n* = 4) (Table 1). Diagnosis of pseudoporphyria was based on clinical and sometimes histological features consistent with porphyria, plus negative porphyrin scan. All patients presented with blistering on hands, some with associated skin fragility and scarring, and many experiencing involvement of other exposed areas i.e. face, legs, arms. Oral medication use was reported in 15 cases, most commonly oral contraceptives and analgesics, with possible relevance to triggering several cases. Three of the four solar urticaria cases were first presentation of the condition. Ross et al. [30] reported a serious case of provocation of previously diagnosed solar urticaria, complicated by loss of consciousness and seizure activity attributed to reduced cerebral perfusion, after 2 min sunbed exposure. Two other cases developed dizziness/faintness or syncope, illustrating potential for systematic reactions to sunbeds in solar urticaria [22]. The search did not identify reactions involving actinic prurigo, chronic actinic dermatitis, hydroa vacciniforme, juvenile spring eruption, polymorphic light eruption, xeroderma pigmentosum and other genophotodermatoses.

### Photoaggravated conditions

The 3 reactions involving a photoaggravated disorder were all in patients with systemic lupus erythematosus (SLE; Table 2). Two were exacerbations of a previously diagnosed condition, and one a new presentation triggered by sunbeds. Fruchter et al. [33] reported a 22-year-old woman with acute onset of cutaneous and systemic features, and investigations consistent with SLE. Another woman presented with a seizure, reduced consciousness, joint swelling and widespread rash; lupus cerebritis was diagnosed, and sunbed exposure deemed the probable cause [34]. All three cases developed systemic symptoms and signs, including arthralgia, arthritis, fever and central nervous system involvement, underscoring the potential for extracutaneous disease flares of SLE following sunbed use [33–55].

### Photosensitivity attributed to systemic medications

Ten of the oral drug reactions involved cutaneous photosensitivity and one case was of retinal phototoxicity (Table 3). A 40-year-old woman experienced blurred vision and retinal lesions after a sunbed session where she had removed the UV-protective goggles for a few minutes. Oral hydrochlorothiazide was thought to have induced her retinal phototoxicity [39]. In the cases of drug-associated pseudoporphyria reported here, the authors were more certain of the role of medication than in those listed in Table 1. This includes a 37-year-old woman who developed pseudoporphyria while taking naproxen and using a sunbed for > 1 year [44]. Nettelblad et al. [41] reported two young women who took psoralen tablets prescribed for other people prior to sunbed use, with the aim of increasing their tan. Both experienced severe phototoxic burns and blistering of exposed sites, one being admitted to a burns unit with ~ 30% body surface area (BSA) de-epithelialisation and ~ 50% BSA blisters and requiring skin grafting. Another case report described a 22-year-old female regular sunbed user who developed a photoexposed bullous erythema within two weeks of starting lamotrigine [42]. This was diagnosed as toxic epidermal necrolysis (TEN) induced by the phototoxic metabolite of lamotrigine. Further, Sever et al. [43] described a 17-year-old patient taking chlorpropamide and tetracycline, who developed severe phototoxic burns after sunbed use.

### Photosensitivity attributed to foods and topical agents

Three foodstuffs containing psoralens were reported to cause photosensitivity reactions after sunbed use: parsnips, celery and lime juice (Table 4). Two cases involved skin contact with parsnips [46], while contact with fresh plants was implicated in a cross-sectional study [47], contact with celery in another cross-sectional study [55], and celery ingestion in one case [53]. This included an investigation into an outbreak of phytophotodermatitis in grocery workers handling celery in Oregon, USA, where the most severe cutaneous reactions occurred in sunbed users [55]. The case-rate in sunbed users was 75% (6 of 8 sunbed users developed cutaneous reactions) versus 33% in non-users, and the celery they were handling showed high psoralen levels. Further, a 22-year-old female bar-worker who used a sunbed after spilling a large quantity of lime juice on her skin, developed erythema and blistering on skin exposed to both lime juice and sunbed; it was concluded that furocoumarins caused her photosensitivity [48].

Five cases of other topical agents (non-food substances) were reported to cause photosensitivity on sunbed exposure. One patient added psoralen obtained from a relative to her bath-water before using a sunbed, and developed extensive erythema and blistering (20% BSA) [49]. Vaporised bergamot oil induced a phototoxic bullous reaction in a 41-year-old woman [52]. Another patient applied rue extract generally before sunbed use, and was admitted to a burns unit with a severe phototoxic reaction affecting 85% BSA; she underwent multiple surgical debridements during her 2 and a half months hospitalisation [54]. A further case had photocontact dermatitis affecting skin exposed to ketoprofen, following sunbed use on two occasions [51]. A photoinduced reaction also occurred to lip tattoo pigment following sunbed use [50].

## Discussion

This comprehensive and systematic review evaluated published reports of photosensitivity reactions occurring following community sunbed use. To our knowledge, this is the first review of this topic. Evidence gathered across included studies indicates that a range of severe photosensitivity reactions occur after sunbed use, from photodermatoses and photoaggravated skin disorders, to systemic drug reactions and photocontact reactions to topical agents. They include six cases of extreme reactions requiring lengthy hospital stays, some requiring skin debridement and/or grafting. First manifestations of SLE and solar urticaria occurred, as well as exacerbations in patients previously diagnosed with these conditions. These findings highlight the need for raised awareness of the potential for photosensitivity reactions with sunbed use.

There was a pronounced sex pattern, only 9% cases being male. Reactions in males comprised photoallergic contact dermatitis, pseudoporphyria and solar urticaria. The ~9 F:1 M ratio may be largely attributable to a higher proportion of females using sunbeds, with a 30 centre European study finding a ratio of > 3:1 in 15 countries [2]. Additionally, certain photodermatoses and photoaggravated conditions are commoner in females, including SLE, PLE and solar urticaria [11, 56–58]. Drug-induced photosensitivity also appears modestly commoner in females [59, 60], while the sex ratio reported in pseudoporphyria is inconsistent [13, 61]. The highest prevalence of reactions was in the 20–29 age group, closely followed by ages 30–39. Two cases were < 18 years, with one 17-year-old reported in the UK in 1988, i.e. before UK legislation was introduced in 2010 banning sunbed use in minors, and a 17-year-old reported in 2010 in Turkey where there are no restrictions for minors [62]. These findings indicate that particular attention should be given to health education of young women.

Pseudoporphyria was the commonest photodermatosis. All patients presented with features similar to PCT, leading to several being initially misdiagnosed, while porphyrin testing was subsequently normal. While some cases were reported as strongly associated with medication (Table 3), others were less so or not taking medication (Table 1). Solar urticaria showed severe reactions to sunbeds, with three cases occurring for the first time following sunbed use. A particularly severe reaction accompanied by systemic involvement including angioedema after sunbed exposure reinforces the risks of exposing extensive skin areas to UVR in this condition [30]. Sunbeds were used for a short period of time in cases of solar urticaria, while cases of pseudoporphyria occurred with repeated use, consistent with the typical patterns of these conditions.

While it is well recognised that UVR can exacerbate the cutaneous manifestations of pre-existing SLE, there are also occasional reports and experimental studies suggesting UVR may exacerbate internal involvement in SLE, and trigger a first manifestation of the condition [63]. Our review notably includes case reports of first manifestations of SLE following community sunbed use [33] and a particularly severe reaction involving a seizure secondary to lupus cerebritis [34]. Our findings thus support that artificial sources of UVR are a precipitating factor for SLE, and highlight the potential life-threatening nature of sunbed use in SLE, as with solar urticaria. There is thus a compelling need for health messages regarding sunbed exposure for people with certain medical conditions. Presently such health messages are patchy, e.g. the USA lupus patient organisation warns tanning beds are unsafe for people with lupus, while the UK organisation presently does not comment [64, 65].

Drug-induced photosensitivity reactions following sunbed use were reported with voriconazole, nalidixic acid, hydrochlorothiazide, lomefloxacin, lamotrigine, psoralen, naproxen, chlorpropamide and tetracycline, most of which, although not all, are well-recognised photosensitisers [66–68]. Even short exposures to sunbeds could induce severe reactions, including TEN, extensive burns and retinal damage. Photodistributed TEN developed in a regular sunbed user shortly after commencing lamotrigine [42], and progressed after sunbed use in a patient taking ibuprofen [40]. Thus the potential for sunbed use to increase the likelihood of such a serious side effect warrants consideration. The occurrence of severe phototoxic burns requiring hospitalisation and skin-grafting after taking psoralen tablets prescribed for other people, prior to sunbed use [41], highlights a hazard of misuse of photosensitising medication. The single ocular reaction identified [39] indicates a further complication of sunbed use; while hydrochlorothiazide is a well-recognised skin photosensitiser [69], it is not well known to affect eyes. These drug-induced photosensitivity reactions, which can be of extreme severity, suggest the need for review of this arena, including improvements in medication labelling and health information.

Psoralens are present in relatively high concentration in plant foodstuffs of the *Rutaceae* (including lime/lemon/grapefruit) and *Umbelliferae* (including celery/parsnip/carrot) families, which were associated with sunbed reactions in this review. One photosensitising reaction was reported following ingestion [53]. This rare occurrence may reflect the highly variable psoralen content of a given plant product, which is dependent on factors including growing location/season and storage length/conditions [16]. More frequently reported was phytophotodermatitis through plant handling, as found in grocery store workers exposed to psoralen-rich plants, where sunbed-users were at substantially higher risk of phytophotodermatitis than non-sunbed-users [47, 55]. Topical psoralens and rue extract led to particularly severe reactions. Rue extract is an extract of *Ruta graveolens*, which contains psoralens and also alkaloids with phototoxic properties; the severe reaction reported following application to the skin (to ‘have good luck’) before using a sunbed [54] highlights the risks of combining commercial tanning and topical applications, such as those perceived to enhance well-being [70].

Strengths of this study include that this is the first study to collate and evaluate published reports of adverse photosensitivity reactions to community sunbed use. It is a comprehensive and systematic review of the literature of this largely overlooked topic, following PRISMA guidelines. As would be anticipated through the nature of this topic, limitations include that the data are primarily from case reports/case series, with the causative role of sunbeds being circumstantial. Within this limitation, application of the appropriate JBI quality assessment tools (Tables 5, 6 and 7) indicated that the majority (28 of 34) of included studies were at low risk of bias for their study design. Moreover, it is well-recognised that case reports and series are a valuable method for detecting adverse reactions [20, 71, 72]. Currently, they provide the best available evidence to inform this topic, and our review addresses a specific information gap. The more severe photosensitivity reactions may present for medical care and are subsequently published, and this is supported by the absence of reports of sunbed-provocation of the commonest and usually milder photodermatosis, PLE. A small number (*n* = 2) of published abstracts were included in order not to miss potentially significant reactions, which may limit the authenticity of this data. It is likely that many cases occur without subsequent publication, such that the cases we present may be a considerable underestimate of the true number. Future research could collect data on unpublished cases, including through surveys of family doctors and hospital emergency departments, to further explore the scope and prevalence of photosensitivity reactions after community sunbed use.

## Conclusions

This study shows the wide-ranging and severe photosensitivity reactions reported after community sunbed use. Several cases required prolonged hospitalisation, and first presentations and exacerbations of significant photodermatoses and photoaggravated conditions occurred, as well as exogenously-triggered reactions to a range of medications, foodstuffs and topical agents. The information provided by this review supports the updating of current guidance on measures to minimise risk of such reactions, such as in the most recent World Health Organisation sunbed report, where little attention is given to photosensitivity reactions other than exogenously triggered, and their potential severity is not recognised [17]. Our findings support the provision of enhanced information and precautions, including for patients with photodermatoses and conditions prone to photoaggravation, such as solar urticaria and SLE, their patient support groups and healthcare professionals, and to prescribers and users of drugs with photosensitising properties. Consideration is warranted to provision of specific training for sunbed-outlet staff and warnings for users at sunbed outlets, regarding the scope and potential severity of photosensitivity reactions.

## Data Availability

Data are provided within the manuscript.
